# Cytomegalovirus Viremia after Living and Deceased Donation in Kidney Transplantation

**DOI:** 10.3390/jcm9010252

**Published:** 2020-01-17

**Authors:** Ulrich Jehn, Katharina Schütte-Nütgen, Joachim Bautz, Hermann Pavenstädt, Barbara Suwelack, Gerold Thölking, Hauke Heinzow, Stefan Reuter

**Affiliations:** 1 Department of Medicine D, Division of General Internal Medicine, Nephrology and Rheumatology, University Hospital of Muenster, 48149 Muenster, Germany; Ulrich.jehn@ukmuenster.de (U.J.); katharina.schuette_nuetgen@ukmuenster.de (K.S.-N.); joachim.bautz@ukmuenster.de (J.B.); hermann.pavenstaedt@ukmuenster.de (H.P.); barbara.suwelack@ukmuenster.de (B.S.); gerold.thoelking@ukmuenster.de (G.T.); 2 Department of Medicine B, Division of Gastroenterology and Hepatology, University Hospital of Muenster, 48149 Muenster, Germany; hauke.heinzow@ukmuenster.de

**Keywords:** kidney transplantation, renal disease, Cytomegalovirus, CMV, Valganciclovir, antiviral prophylaxis, living donation

## Abstract

Despite screening, effective anti-viral drugs and risk-balanced prophylaxis, cytomegalovirus (CMV) remains a major cause of morbidity in transplant patients. The objective of this study was to retrospectively analyze the risk factors associated with CMV viremia after kidney transplantation in a large European cohort with standardized valganciclovir prophylaxis in the present era. A special focus was placed on the comparison of living and postmortal donation. We conducted a longitudinal observational study involving 723 adult patients with a total of 3292 patient-years who were transplanted at our center between 2007 and 2015. Valganciclovir prophylaxis was administered over 100 days for CMV+ donors (D) or recipients (R), over 200 days for D+/R−, and none in D−/R−. A CMV+ donor, rejection episodes, and deceased donor transplantation were identified to be associated with increased incidences of CMV viremia. Although we did not find a reduced overall survival rate for patients with CMV viremia, it was associated with worse graft function. Since we observed a relevant number of CMV infections despite prescribing valganciclovir prophylaxis, a pre-emptive strategy in patients with (suspected) adherence restrictions could be favored. Our data can help transplant physicians educate their patients about their individual CMV risk and choose the most appropriate CMV treatment approach.

## 1. Introduction

Cytomegalovirus (CMV) is the most prevalent pathogen in transplant patients. Therefore, CMV infection is still an issue after kidney transplantation (KTx) [1]. Since CMV viremia can occur as a result of a reactivation of a latent infection or result from a primary infection when, for example, it is transmitted from an infected donor through a kidney transplant, it is not surprising that CMV viremia frequently (re-)occurs after KTx [2,3]. The level of endemic CMV seroprevalence ranges from about 40%–50% in highly developed countries to almost 95% in developing countries [4]. CMV seropositivity does not exclude donation, as CMV infection prophylaxis and therapy are available [2]. However, a primary CMV infection is considered more serious than a reactivation, exposing seronegative recipients of seropositive organs to the highest risk [2]. Other risk factors for CMV infection include the intensity of the immunosuppression, i.e., the administration of lymphocyte-depleting antibodies, critical illness or co-infection, leuko- and lymphopenia, as well as genetic polymorphisms and cold ischemia time [4]. Ultimately, the degree of impairment of T–cell-function determines the incidence of CMV disease [5]. In patients at increased risk of transmission, surveillance and/or prophylaxis strategies are part of the standard care after kidney transplantation [3]. These strategies are effective to reduce CMV-associated disease burden, but despite antiviral prophylaxis, up to 60% of KTx patients develop CMV viremia, and approximately 20% even develop symptomatic CMV disease [6,7].

In addition to an acute (organ) infection, CMV can affect the immunity by chronic stimulation of the immune system, as it cannot be eliminated even by healthy persons. Recurrent episodes of subclinical reactivation in immunocompromised patients foster an immune exhaustion with consecutive opportunistic infections [5]. In addition, indirect effects related to CMV infection include acute and chronic rejection as well as transplant vasculopathy [8].

The prophylaxis of CMV infection in kidney recipients at risk is now routinely carried out with antiviral agents such as ganciclovir or valganciclovir. However, the strategies used differ [9]. As an alternative, mechanistic Target of Rapamycin (mTOR)-inhibitor-based immunosuppression could reduce the incidence of CMV infections in high-risk patients [10].

Another approach is a pre-emptive CMV therapy guided by a regular polymerase chain reaction (PCR)-based virus monitoring, which turned out to be equally effective in the prevention of serious complications of CMV infections [11]. 

We herein aimed to identify risk factors for CMV DNAemia (hereafter referred to as viremia) and their implications for transplant dysfunction and overall survival in the present era [12].

## 2. Materials and Methods

### 2.1. Study Design and Population

We conducted a longitudinal observational study involving 723 adult patients with a total of 3292 patient-years who were transplanted at our center between 2007 and 2015. The demographic and clinical characteristics of patients were assessed at the time of KTx. Informed written consent from all patients was obtained to record their data at the time of transplantation. Prior to the analysis, the patient data was anonymized. This study was performed in accordance with the Declaration of Helsinki and the International Conference on Harmonization Good Clinical Practice guidelines and approved by the local ethics committee (Ethik Kommission der Ärztekammer Westfalen-Lippe und der Medizinischen Fakultät der Westfälischen Wilhelms-Universität, 2014-381-f-N). The data was extracted from the patients’ files. The induction therapy was chosen according to the immunological risks of the recipient independently from the donor status (standard or extended criteria donor) (Table 1). One g mycophenolate mofetil was given bis in die, and the dosage was reduced in case of adverse events. Prednisolone was started with 500 mg intravenously before KTx, followed by 100 mg for three days, and then reduced by 20 mg/day. A dosage of 20 mg/day was maintained until day 30 and then slowly reduced to 5 mg/day. Immunosuppressive maintenance therapy usually consisted of a calcineurin inhibitor (tacrolimus or cyclosporine A), mycophenolate sodium or mycophenolate mofetil (MMF), and prednisolone. M-TOR-inhibitor-based immunosuppression after KTx was only chosen in a minority of patients and only when they were included in clinical trials.

### 2.2. CMV

Oral CMV-prophylaxis with valganciclovir was administered over 100 days for donors (D+)/recipients (R−), D+/R+, and D−/R+ recipients, over 200 days for D+/R− recipients beyond June, 2009, and none if both the donor and the recipient were negative for CMV [13]. CMV viremia (considered relevant if >214.6 copies/mL corresponding to the threshold value given by the manufacturer (90% CI 163 to 355 IU/mL) was documented. CMV screening was performed monthly within the first 6 months after KTx, every second month during months 6–12, and on indication. Ethylenediaminetetraacetic acid (EDTA)-blood was collected from patients to assess CMV DNA by polymerase chain reaction using the kPCR PLX^®^ CMV DNA-Assay in combination with the VERSANT^®^ kPCR molecular system (Siemens Healthcare Diagnostics, Eschborn, Germany).

### 2.3. Statistical Analysis

The data was analyzed with IBM SPSS Statistics 24^®^ (IBM Corp., Armonk, NY, USA). The results are expressed as a median with interquartile range (IQR), or number/percent. Non-continuous parameters were analyzed by Fisher’s exact test and chi-square test and continuous parameters were analyzed by the Mann-Whitney U-test and Kruskal–Wallis test respectively, if appropriate. A *p*-value below 0.05 was considered statistically noticeable. 

The cumulative probability of developing CMV infection in the kidney transplant cohort was calculated by Kaplan–Meier analysis and the curves were compared using the log-rank test. The cumulative incidence of the first CMV diagnosis at one, three, and five years of follow-up was calculated.

Univariate analysis to identify potential predictors of CMV development was performed. Only variables that were considered statistically noticeable by univariate analysis were used for multivariable analysis to identify independent prediction factors for CMV development.

## 3. Results

Baseline characteristics of the study populations are given in Table 1. Median age at the transplantation was 53 years (range 41–66), 60.4% were male, and 28.6% received a living donor transplantation.

Usually, induction therapy with basiliximab (84%) was performed, 5% of the patients received thymoglobuline (Table 1).

### 3.1. CMV Viremia

**Figure 1:**
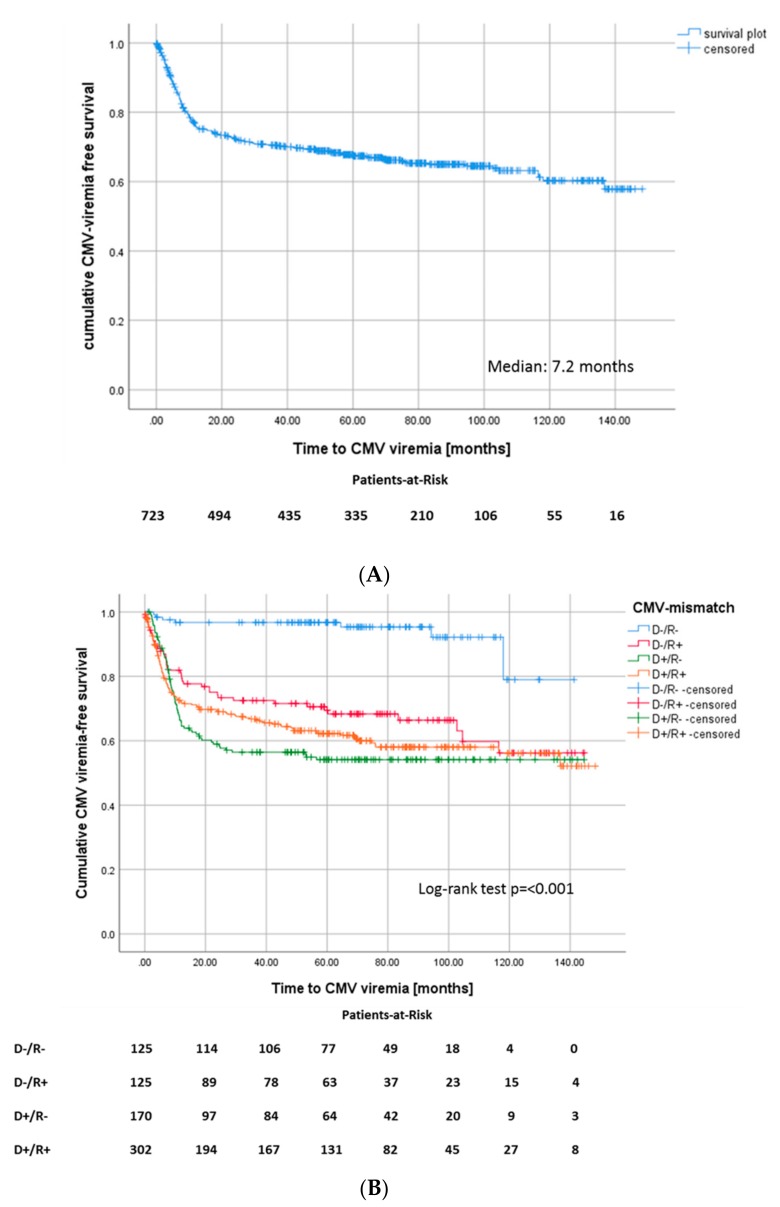
(**A**) Kaplan–Meier survival plot for onset of CMV viremia after transplantation, (Median: 7.2 months) and (**B**) for onset of CMV viremia after transplantation according to CMV match.

Of all patients, 239 (33.1%) developed at least one episode of proven CMV viremia. The median time until onset of CMV was seven months (Figure 1). The cumulative incidence of CMV viremia at one, three, and five years was 23.1%, 28.4%, and 30.7% respectively, resulting in an incidence of 7.3 infections per 100 person-years. Of all CMV-viremia episodes, 19.2% were detected within the first 100 days after transplantation, when antiviral prophylaxis should have been maintained in most patients.

### 3.2. Association between CMV Viremia, CMV Mismatch, and Outcome Parameters after KTx

Of the 723 study participants, 105 (14.5%) died before the end of our observation period, with a mean age of 65.0 ± 11.5 years at the time of death. Thirty-four (32.4%) of the deceased patients died by reason of a cardiovascular event, 27 (25.7%) due to infection, 24 (22.9%) due to malignancy, and 2 (1.9%) because of other reasons. In 18 cases (17.14%), the cause of death was unknown. 

**Figure 2:**
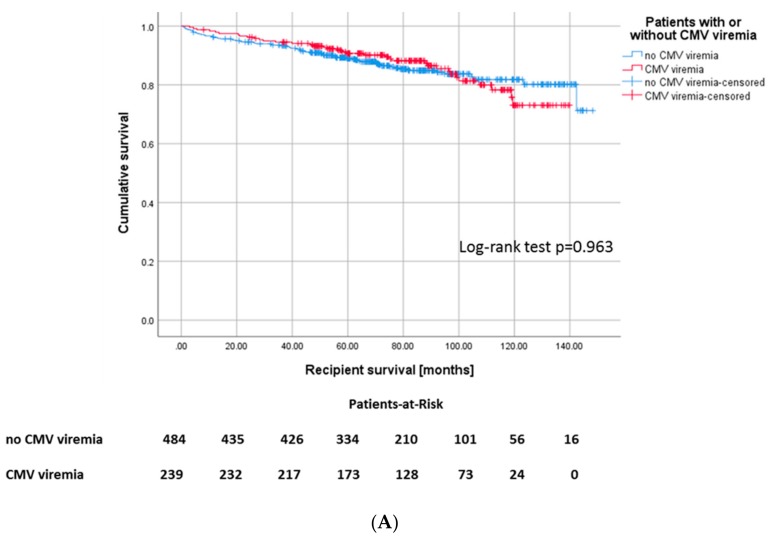

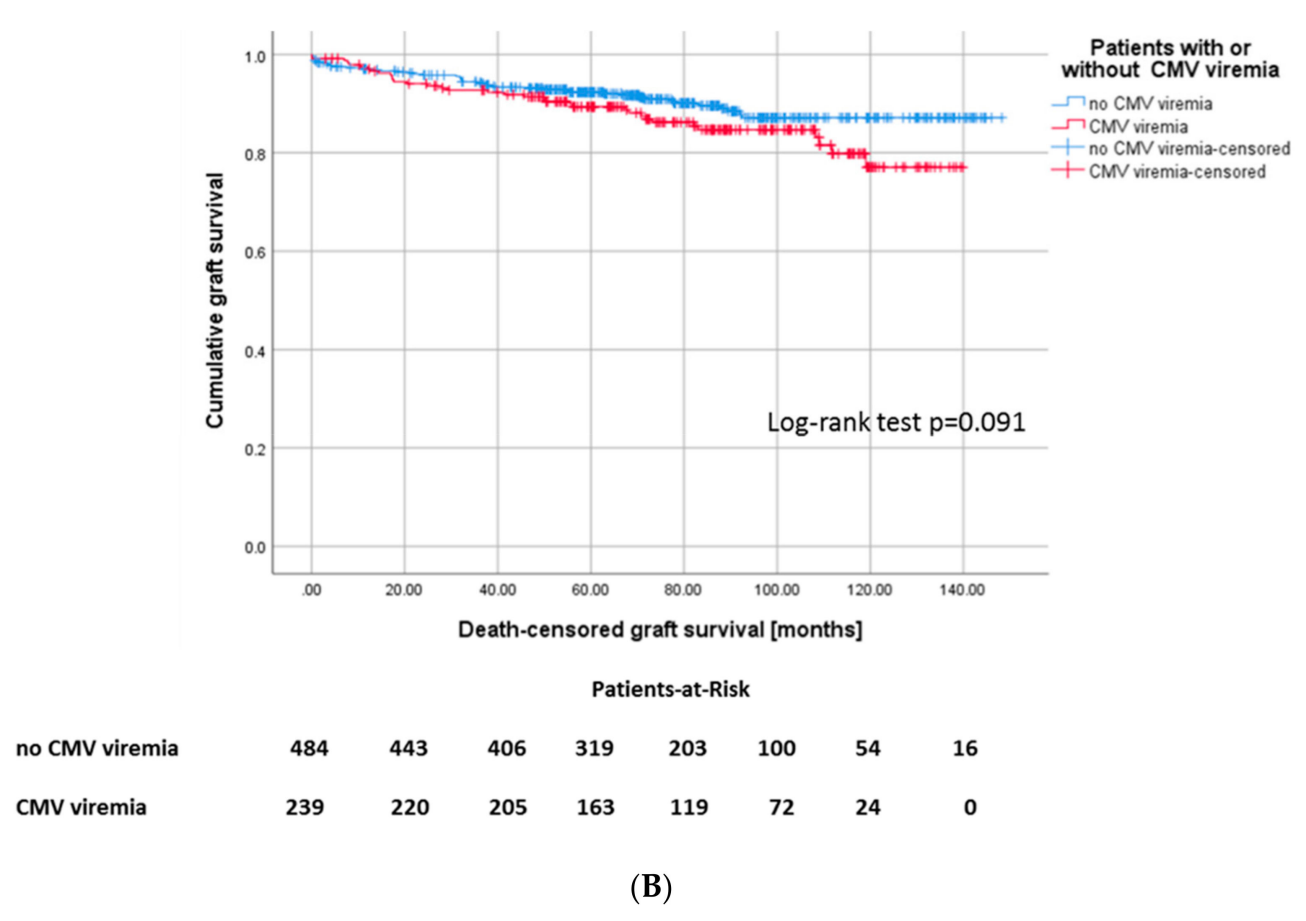
(**A**) Kaplan–Meier plot for recipient survival, Log-rank: *p* = 0.444 and (**B**) death-censored graft survival, *p* = 0.091 according to development of CMV infection.

The overall survival of recipients in the CMV+ cohort was not different from the CMV− cohort (*p* = 0.963, Figure 2A). Likewise, the overall graft survival did not differ between CMV+ and CMV− patients (Figure S1).

Patients who developed CMV infection showed a noticeably lower estimated glomerular filtration rate (eGFR) after one year (48.8 versus 55.2 mL/min/1.73 m^2^, *p* = 0.000), after three (50.2 versus 54.6 mL/min/1.73 m^2^, *p* = 0.008), and five years (47.5 versus 53.2 mL/min/1.73 m^2^, *p* = 0.002), respectively (Table 2).

Additionally, patients who developed CMV viremia had a noticeably higher proteinuria after one year (278 mg/g creatinine versus 231 mg/g creatinine, *p* = 0.001) (Table 2).

However, the incidence of terminal graft failure was not different between the groups, although there was a tendency towards a longer graft survival in the CMV− cohort (135 ± 1.8 months versus 122 ± 2.7 months, *p* = 0.091, Figure 2B).

**Figure 3:**
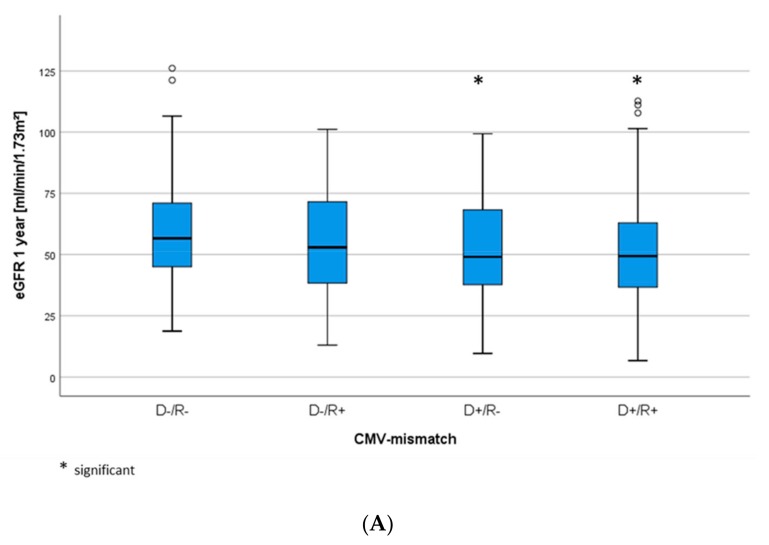

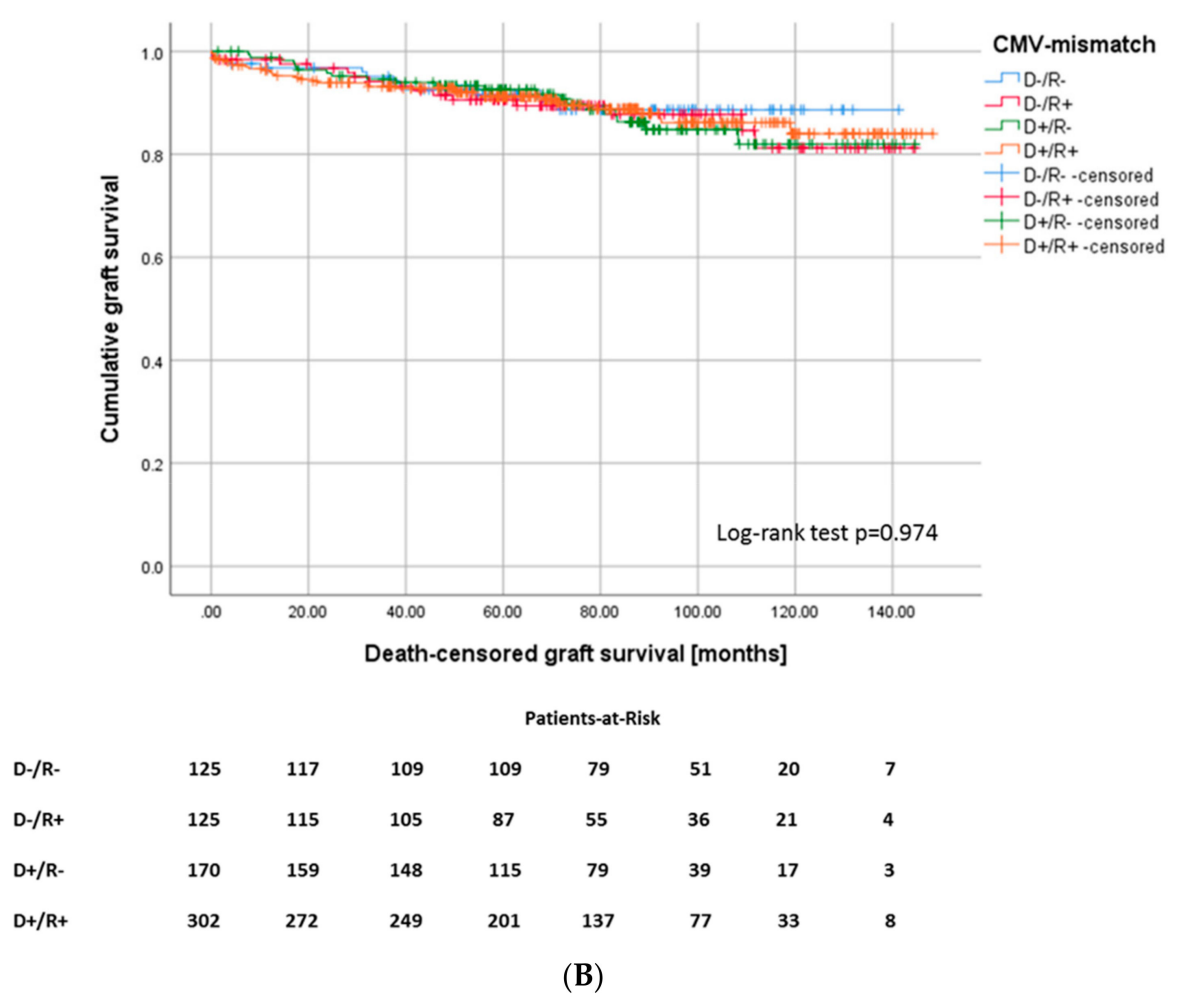
(**A**) eGFR levels after one year and (**B**) death-censored graft survival (Log-rank *p* = 0.974), according to CMV-mismatch.

Notably, in addition to CMV viremia, pure CMV mismatch may already determine a worse graft function. The Kruskal–Wallis test shows a noticeably lower eGFR after one year for intermediate-high and high-risk patients compared to low- and intermediate-risk patients (*p* = 0.014) (Figure 3A). Nevertheless, death-censored graft survival did not differ between the CMV risk groups (Figure 3B).

**Figure 4:**
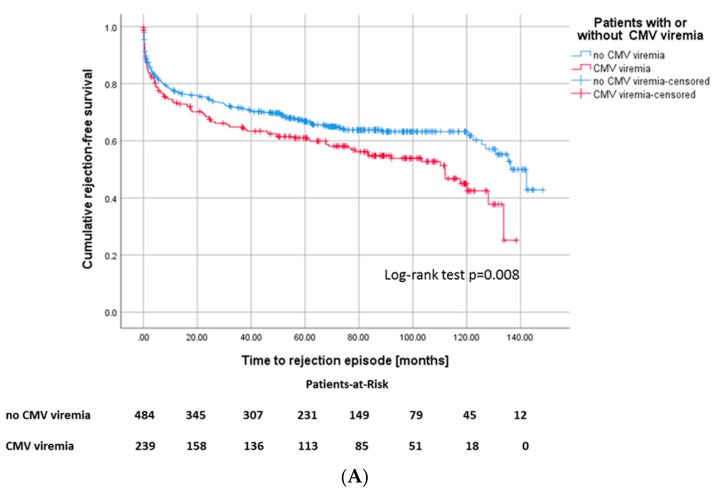

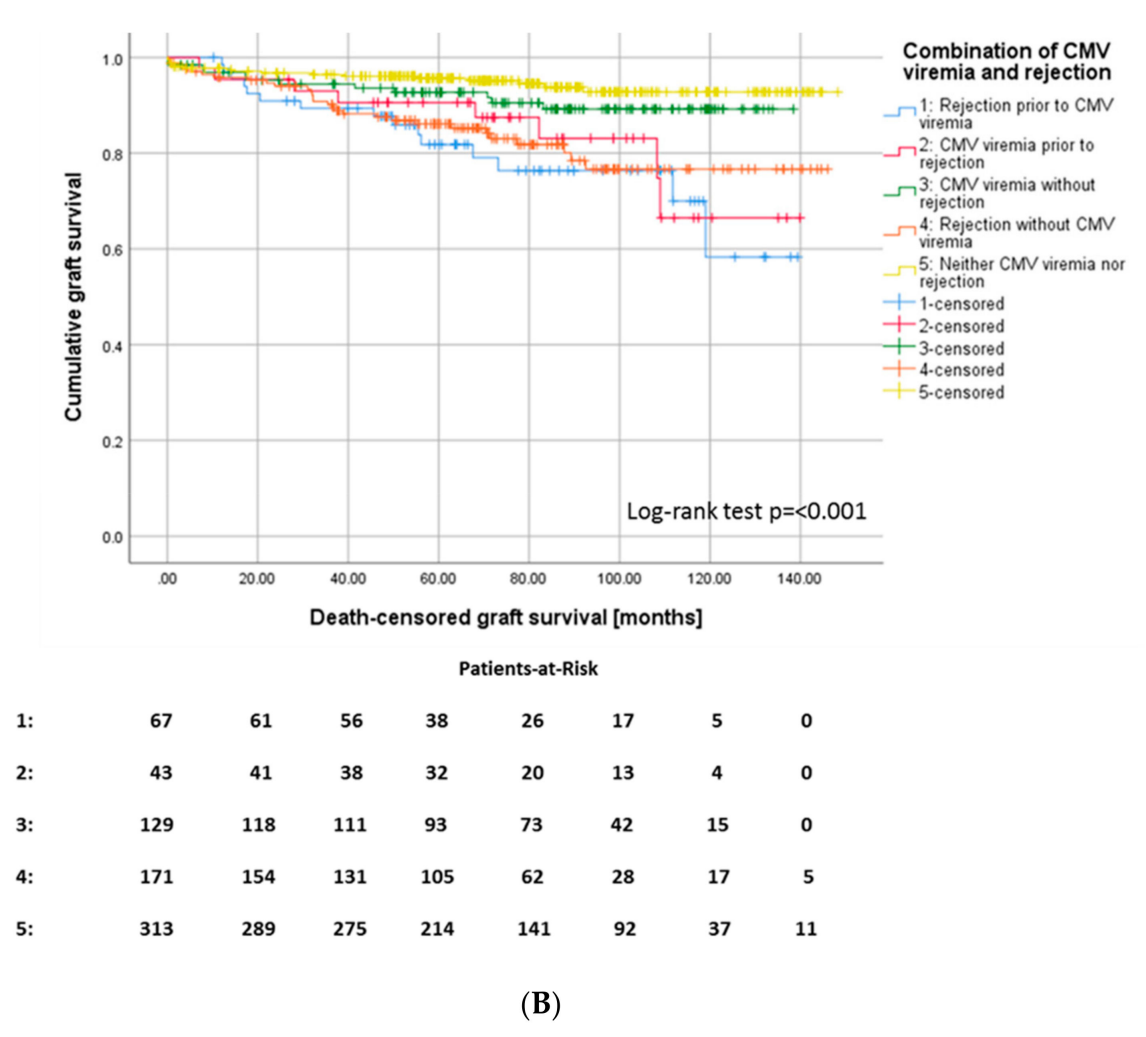
(**A**) Kaplan–Meier survival plots for the incidence of rejection episodes, *p* = 0.008 according to CMV viremia, and (**B**) for death-censored graft survival according to the different constellations of onset of CMV viremia and rejection episodes, *p* ≤ 0.001.

In our patient cohort, 282 (39%) patients had at least one biopsy-proven rejection episode (Table 2). Those recipients with CMV viremia featured a higher risk for any type of rejection episode, 46% of CMV+ recipients sustained at least one rejection episode in the course, whereas only 35.3% of CMV– recipients underwent rejection (Figure 4A). In 67 (60.4%) of 110 patients in total with both CMV viremia and rejection, rejection occurred before CMV viremia in 44 patients (39.6%) after occurrence of CMV viremia (Figure 4B). 171 recipients of the cohort (23.7%) developed a rejection without diagnosis of CMV viremia.

**Figure 5:**
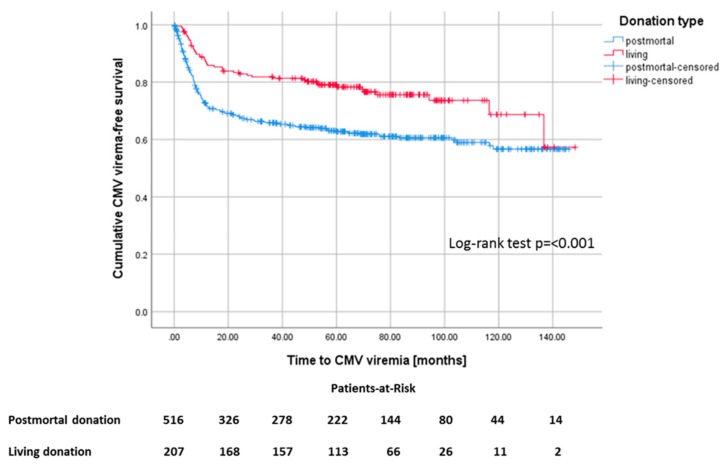
Kaplan–Meier survival plot for the development of CMV viremia, *p* ≤ 0.001, according to living versus postmortal donation.

Recipients of a deceased-donor kidney transplant had a higher risk of developing CMV viremia with a mean onset time of 93 months (CI 87.1–98.9) after KTx compared to 114 months after living donations (CI 105.2–122.2) (Figure 5). In our study cohort, 207 (28.6%) of the patients received a living donation, 41 (19.8%) of whom were AB0-incompatible. Living donation was associated with younger age of donor and recipient, shorter cold and warm ischemia times, lower incidences of delayed graft function (DGF) and New-onset diabetes after transplantation (NODAT), and shorter time on dialysis compared to postmortal donations. For postmortal donations, the total number of human leukocyte antigen (HLA)-mismatches and the proportion of CMV low-risk patients was noticeably lower (Table 3).

**Figure 6:**
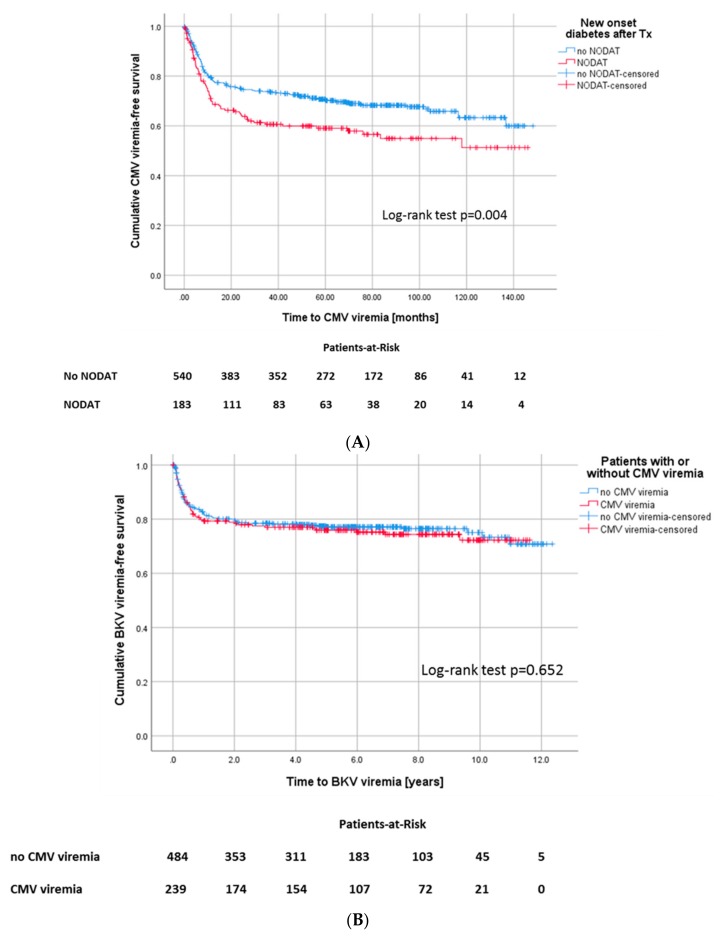
(**A**) Kaplan–Meier survival plot for onset of CMV viremia, according to development of NODAT, Log-rank test: *p* = 0.004 and (**B**) for onset of BKV viremia, Log-rank: *p* = 0.652, according to development of CMV infection.

In patients who developed a CMV viremia, the incidence for new onset diabetes after transplantation (NODAT) was noticeably increased (*p* = 0.018) (Figure 6A). In patients with D−/R− serostatus, the incidence of NODAT was noticeably lower (20.2%) compared to D+/R− (39.4%), D+/R+ (36.0%), and D−/R+ (37.4%) (*p* ≤ 0.001).

168 of all patients (23.2%) were diagnosed with BK-polyomavirus (BKV) viremia. In detail, 22% of patients in the CMV-negative group developed BKV viremia, 24.7% of CMV-positive patients also developed BKV viremia in the course. Statistically, there was no difference between the groups (*p* = 0.514) (Figure 6B).

A delayed graft function was more frequently diagnosed in patients who sustained CMV viremia later on (30.5% versus 21.3%, *p* = 0.018).

### 3.3. Identified Risk Factors Associated with CMV Viremia

Patients who developed a CMV infection in the follow-up had a mean age of 54.4 years compared to patients without CMV, who were 50.8 years old on average. Cold ischemic time was not different between patients with and without CMV viremia (8.5 versus 8.1 h), although there was a noticeably lower number of living donor transplants in the CMV− group (32.6% versus 20.5%). Analysis of the CMV match revealed a higher proportion of patients with a high-risk constellation (D+/R−) 31.4% versus 19.6% and intermediate-high risk (D+/R+) 48.5% versus 38.4% constellation in the cohort with CMV viremia. Accordingly, the low-risk constellation (D−/R−) 2.9% versus 24.4% was more prevalent in the CMV− cohort. The incidence of delayed graft function (DGF, defined as the need for dialysis within one week after KTx) (30.5% versus 21.3%) as well as the occurrence of NODAT (31.5% versus 22.5%) was noticeably increased in the CMV+ cohort.

In binary logistic regression analysis, CMV match, age of recipient at Tx, age of the donor, NODAT, DGF, initial use of MMF, and number of rejections were identified to be noticeably associated with increased occurrence of CMV viremia (Table 4). Living donation and the use of cyclosporine A instead of tacrolimus were protective with respect to the onset of CMV viremia (Table 2). 

Multivariable regression analyses verified that CMV match, age of the donor, and number of rejections correlated negatively with the incidence of CMV after KTx (Table 5), whereas living donation was confirmed to be protective.

## 4. Discussion

Our study focused on the association of recipient, donor, and transplant variables with the onset of CMV viremia and its outcomes in the present era.

Consistent with other studies, the vast majority (70%) of CMV infections in our cohort occurred within the first year after transplantation. In addition, the proportion of transplanted patients, who developed a CMV viremia, is consistent with the results from other studies [11,14]. 

In 19.2% of patients in our CMV+ cohort, viremia emerged within the first three months after transplantation, probably promoted by the higher initial immunosuppression. Notably, during this time, patients at risk should have been treated with antiviral prophylaxis in accordance with our center guidelines. Multivariate analysis revealed a strong association for the onset of CMV viremia with CMV-mismatch. The higher risk of CMV infection for D+/R− and D+/R+ serostatus (*p* ≤ 0.001) confirms the results of several other studies (e.g., References [6,15]). The incidence of CMV viremia differed between CMV low risk patients and those on antiviral prophylaxis already during the first three months (Figure S2). 

Three possible explanations are conceivable. First, a significant lack of adherence to the medication across the patients in the different CMV categories, second, an under-dosing or stop of medication due to adverse effects, or third, the occurrence of valganciclovir-resistant CMV strains. Since we did not observe a high percentage of valganciclovir-resistant CMV infection in our center, the other explanations might be favored. This raises the question of the reasonability of the prophylaxis taking the adverse effects, the development of drug resistance, and costs of the virostatic medication into account. This leads to the current ongoing discussion about preemptive CMV therapy instead of prophylaxis. Some studies demonstrate that preemptive therapy is at least as effective as prophylactic therapy in terms of survival of patients and grafts [11,16]. In contrast, the results of a retrospective study in patients at a high risk of CMV infection (D+/R−) support the prolongation of antiviral prophylaxis with low-dose valganciclovir (450 mg daily) in these patients, up to 12 months. It has been observed that this approach is associated with a lower CMV infection rate and an almost eradication of late-onset CMV disease [17]. Interestingly, the median time of CMV onset in our cohort was approximately seven months, just after the end of the 200-day prophylaxis of the D+/R− group. In summary, the implementation of a preemptive strategy does not appear to be detrimental in patients with a tendency to limited adherence or adverse effects to valganciclovir.

In contrast to other studies, we did not find a noticeably reduced graft survival and overall survival in the CMV+ cohort (Figure 2), although these patients were 3.5 years older at transplantation [14,18,19,20]. One may speculate that increased CMV surveillance and early treatment could be reasons for this observation in the present era. Further, there was only a tendency for inferior graft survival in the CMV+ cohort (13 months less), despite noticeably higher incidences of DGF, NODAT, and rejections in the CMV+ cohort. Nevertheless, patients with CMV viremia had worse allograft function one, three, and five years after KTx. 

In accordance with a study by Santos et al. [21], CMV serology was associated with the incidence of NODAT. D−/R− had a noticeably lower risk for the development of NODAT compared to D+/R−, D+/R+, and D−/R+, who had an equal risk.

Deceased donor transplant was identified in the multivariable analysis to be noticeably associated with CMV viremia, as living donation had an odds ratio of 0.54 for the development of CMV viremia (*p* = 0.005). This is consistent with the literature [14,22]. Further associations found were the age of the donor (*p* = 0.004) and the number of rejection episodes (*p* ≤ 0.001), while the use of cyclosporine A was protective (*p* = 0.024). Usually, living donations are characterized by younger donors and recipients, a shorter dialysis vintage, as well as shorter cold and warm ischemia periods leading, for example, to lower DGF rates. In contrast, HLA matching was worse in this group (Table 3). As CMV positivity increases with lifetime, the younger donor age was associated with a lower proportion of CMV+ donors [14]. Nevertheless, CMV match and postmortal donation were both detected as independently associated with CMV viremia in multivariable analysis.

Both acute and chronic rejections can be related to CMV infection [23]. On the one hand, several studies have shown that CMV increases the risk of allograft rejection (e.g., by fostering an immune dysbalance or activation of graft specific immune reactions), but on the other hand, increased immunosuppression in the event of rejection increases the risk for CMV [24,25]. In line with this, weaker immunosuppression with cyclosporine A or m-TOR instead of tacrolimus was associated with less CMV infections. The role of immunosuppression was emphasized by Felipe et al., who even observed that previous acute rejection episodes were the major risk factor for CMV infection in the first year after transplantation [26]. Notably, we made both observations as in 60.4% of the cases CMV viremia occurred after rejection, whereas in 39.6% of patients, CMV viremia occurred before rejection. Multivariate analysis confirmed that episodes of acute rejection are associated with the development of CMV viremia. However, 54% of CMV viremias were not related to rejections. 

The relationship between CMV viremia and BK viremia remains controversial [27,28]. We did not notice an association between CMV and the occurrence of BK viremia. Interestingly, there is data suggesting that BK viremia is related to valganciclovir prophylaxis [29]. However, we could not observe this association [30].

A recently published study by Leeaphorn et al. demonstrates in a large patient cohort that a mere CMV mismatch with regard to constellations with high and intermediate high-risk has a negative impact on the graft survival [31]. In our cohort, CMV-mismatch in terms of high and intermediate high-risk indeed determines lower eGFR rates, though without impacting graft survival.

Our study has limitations. First, because it is a retrospective analysis, the study can only generate hypotheses. Second, the amount of CMV load was not considered. This could be relevant, because a study by Reischig et al. showed an increased risk of graft loss in patients with higher viral load [32], and Blazquez-Navarro et al. found a significant GFR-impairment for CMV-viral loads of >10,000 copies/mL [33]. Third, we have no data on the adherence of patients or the adverse effects of valganciclovir. Fourth, we have not differentiated between asymptomatic CMV viremia and invasive CMV disease. In other study cohorts, approximately 75% of patients with CMV viremia present symptoms of CMV disease (e.g., Reference [34]). Invasive CMV disease can worsen the all-cause mortality and graft loss outcome [35].

This study is of interest because we provided a comprehensive analysis of the factors associated with CMV viremia after living as well as postmortal kidney donation in a large European KTx cohort with long-term follow-up using a standardized valganciclovir prophylaxis. Our data can help transplant physicians educate their patients about their individual CMV risk and choose the most appropriate CMV treatment approach.

## 5. Conclusions

Despite prophylaxis, CMV viremia is still a relevant infection after KTx, affecting nearly every third patient within the first year after transplantation, especially after the end of the prophylaxis. We observed a relevant number of CMV viremia despite prescribing valganciclovir prophylaxis. Further studies will have to examine whether a preemptive strategy could be beneficial in patients with (suspected) compliance restrictions. A CMV+ donor, rejection episodes, and deceased donor transplantation were identified to be associated with increased incidence of CMV viremia. Although we did not observe a reduced overall survival for patients who developed a CMV viremia, CMV was associated with worse graft function and a tendency towards a 13 months shorter graft survival.

## Figures and Tables

**Table 1:** Patients’ demographic and clinical characteristics at transplantation.

Variable	Non-CMV	CMV	*p* Value
**Patients (*n*)**	484 (68.2%)	239 (33.1%)	**0.000 ^a^**
Age at Tx * (years)	50.8 ± 13.9	54.35 ± 14.1	**0.010 ^a^**
**Sex male (*n*)**	300 (62%)	137 (57.3%)	**0.258 ^b^**
ABO incompatible Tx * (*n*)	30 (6.2%)	11 (4.6%)	**0.494 ^b^**
**mismatch-HLA-A**			**0.838 ^b^**
none	173 (35.7%)	80 (33.5%)	
1	228 (47.1%)	117 (49%)	
2	81 (16.7%)	41 (17.2%)	
**mismatch-HLA-B**			**0.925 ^b^**
none	113 (23.3%)	55 (23%)	
1	232 (47.9%)	112 (46.9%)	
2	137 (28.3%)	71 (29.7%)	
**mismatch-HLA-DR**			**0.482 ^b^**
none	129 (26.7%)	56 (23.4%)	
1	231 (47.7%)	113 (47.3%)	
2	122 (25.2%)	69 (28.9%)	
**PRA >85% (*n*)**	61 (12.6%)	26 (10.9%)	**0.539 ^b^**
**PRA >5% (*n*)**	10 (2.1%)	7 (2.9%)	**0.447 ^b^**
Living donor Tx * (*n*)	158 (32.6%)	49 (20.5%)	**0.001 ^b^**
**Cold ischemia time (hours)**	8.1 ± 5.3)	8.53 ± 4.76	**0.175 ^a^**
**Warm ischemia time (minutes)**	32.7 ± 8.2	33.5 ± 8.5	**0.332 ^a^**
Dialysis prior to Tx * (*n*)	443 (91.5%)	228 (95.4%)	**0.054 ^b^**
**Time on dialysis (months)**	57.7 ± 43.7	56.8 ± 36.8	**0.747 ^a^**
Previous Tx * (*n*)	58 (12%)	36 (15.1%)	**0.29 ^b^**
**CMV mismatch D/R**			**0.000 ^b^**
D−/R−	118 (24.4%)	7 (2.9%)	
D−/R+	84 (17.4%)	41 (17.2%)	
D+/R−	95 (19.6%)	73 (31.4%)	
D+/R+	186 (38.4%)	116 (48.5%)	
**Induction therapy**			**0.543 ^c^**
No induction therapy	15 (3.1%)	12 (5%)	
Basiliximab induction (*n*)	407 (84.1%)	198 (82.8%)	
Thymoglobulin (*n*)	21 (4.4%)	16 (6.7%)	
Alemtuzumab (*n*)	9 (1.9%)	5 (2.1%)	
Eculizumab (*n*)	2 (0.4%)	1 (0.4%)	
Rituximab (*n*)	30 (6.2%)	10 (4.2%)	
**Initial steroid use**	473 (97.8%)	235 (98.3%)	**0.758 ^b^**
Initial MMF * use	456 (94.2%)	235 (98.3%)	**0.039 ^b^**
Initial CyA * use	21 (4.4%)	2 (0.8%)	**0.007 ^b^**
**Initial tacrolimus use (*n*)**	459 (94.8%)	237 (99.2%)	**0.007 ^b^**
Initial mTOR * inhibitor use (*n*)	24 (5.0%)	5 (2.1%)	**0.071 ^b^**
**Diagnosis of ESRD, (*n*)**			**0.522 ^c^**
Hypertension	34 (7%)	24 (10%)	
Diabetes	27 (5.6%)	16 (6.7%)	
Polycystic kidney disease	75 (15.8%)	30 (12.6%)	
Obstructive Nephropathy	23 (4.8%)	12 (5%)	
Glomerulonephritis	160 (33.1%)	71 (29.7%)	
FSGS *	18 (3.7%)	15 (6.3%)	
Interstitial nephritis	22 (4.5%)	15 (6.3%)	
Vasculitis	17 (3.5%)	6 (2.5%)	
Other	69 (14.3%)	35 (14.6%)	
Unknown	39 (8.1%)	15 (6.3%)	

^a^ Mann–Whitney U-test; ^b^ Fisher’s exact test; ^c^ Chi square test; * Abbreviations: CMV: cytomegalovirus; Tx: transplantation; HLA: human leukocyte antigen; PRA: panel reactive antibodies; MMF: mycophenolate mofetil; CyA: cyclosporine A; mTOR: mechanistic target of rapamycin; ESRD: end-stage renal disease; FSGS: focal segmental glomerulosclerosis. Bold: Main variables and *p*-values.

**Table 2:** Clinical outcome parameters.

Variable	Non-CMV	CMV	*p* Value
**Time until CMV viremia in months**		16.6 ± 23.9	**-**
**DGF (*n*)**	103 (21.3%)	73 (30.5%)	**0.018 ^b^**
**eGFR at year 1 (mL/min/1.73 m^2^)**	55.2 ± 21.0	48.8 ± 19.8	**0.000 ^a^**
**eGFR at year 3 (mL/min/1.73 m^2^)**	54.7 ± 20.3	51.2 ± 20.9	**0.008 ^a^**
**eGFR at year 5 (mL/min/1.73 m^2^)**	51.2 ± 20.6	47.4 ± 20.7	**0.002 ^a^**
**UPCR at year 1 (mg/g crea)**	231 ± 503	278 ± 339	**0.001 ^a^**
**UPCR at year 5 (mg/g crea)**	392 ± 544	368 ± 496	**0.831 ^a^**
**Overall graft survival (mean, months) (95% CI)**	124.3 (119.8–128.8)	114.8 (108.8–120.7)	**0.444 ^c^**
NODAT * (*n*)	109 (22.5%)	74 (31.5%)	**0.018 ^b^**
**BK viremia**	109 (22.5%)	59 (24.7%)	**0.514 ^b^**
**Rejection yes (*n*)**	171 (35.3%)	110 (46%)	**0.006 ^b^**

^a^ Mann–Whitney U-test; ^b^ Fisher’s exact test; ^c^ Log-rank test; * Abbreviations: CMV: cytomegalovirus; DGF: delayed graft function; eGFR: estimated glomerular filtration rate; UPCR: urine protein/creatinine ratio; NODAT: new onset diabetes after transplantation; BK: BK polyomavirus. Bold: variables and *p*-values.

**Table 3:** Characteristics of living versus postmortal donations.

Donation Type	Living	Postmortal	*p* Value
**Patients (*n*)**	207 (28.6%)	516 (71.4%)	**<0.001 ^a^**
Recipient Age (years) *	43.6 (±13.9)	55.4 (±12.6)	**<0.001 ^a^**
ABO incompatible Tx * (*n*)	41 (19.8%)	0 (0%)	**<0.001 ^b^**
**PRA >5% (*n*)**	23 (11.1%)	62 (12.0%)	**0.799 ^b^**
**Cold ischemia time (hours)**	2.3 ± 0.5	10.7 ± 4.0	**<0.001 ^a^**
**Warm ischemia time (minutes)**	30.7 ± 10.3	33.8 ± 7.2	**<0.001 ^a^**
**Time on dialysis (months)**	20.0 ± 23.9	72.5 ± 37.5	**<0.001 ^a^**
Preemptive Tx * (*n*)	48 (23.2%)	0 (0%)	**<0.001 ^b^**
**Donor age (years)**	51.7 ± 9.4	54.2 ± 16.3	**0.002 ^a^**
DGF * (*n*)	12 (5.8%)	164 (31.8%)	**<0.001 ^b^**
NODAT * (*n*)	32 (15.5%)	151 (29.3%)	**<0.001 ^b^**
**HLA mismatch (0–6)**	3.4 ± 1.5	2.7 ± 1.7	**<0.001 ^c^**
**CMV mismatch (D/R)**D−/R−	52 (25.1%)	73 (14.1%)	**0.003 ^b^**
D−/R+	26 (12.6%)	99 (19.2%)	
D+/R−	48 (23.2%)	122 (23.6%)	
D+/R+	81 (39.1%)	221 (42.8%)	

^a^ Mann–Whitney U-test; ^b^ Fisher’s exact test; ^c^ Chi square test; * Abbreviations: Tx: Transplantation; HLA: Human leukocyte antigen; PRA: Panel reactive antibodies; DGF: delayed graft function; NODAT: New onset diabetes after transplantation. Bold: main variables and *p*-values.

**Table 4:** Univariate binary logistic regression.

Variable	Regression-Coefficient	Odds Ratio	95% CI	*p* Value
**Age at KTx**	0.018	1.019	1.007–1.030	**0.002**
**Previous KTx**	0.264	1.303	0.832–2.093	**0.248**
**CMV Match**	0.456	1.577	1.353–1.838	**<0.001**
**Living Donation**	−0.631	0.532	0.369–0.768	**0.001**
**Donor age**	0.028	1.028	1.016–1.046	**<0.001**
**Mode of dialysis**	−0.110	0.896	0.746–1.076	**0.240**
**Time of dialysis**	0.000	1.000	0.996–1.003	**0.799**
**Cold ischemia time**	0.016	1.016	0.986–1.048	**0.296**
**Warm ischemia time**	0.011	1.011	0.912–1.030	**0.270**
**Highest PRA**	−0.002	0.998	0.991–1.006	**0.683**
**Etiology of kidney failure**	−0.020	0.980	0.924–1.039	**0.500**
**NODAT**	0.434	1.543	1.040–2.154	**0.014**
**Delayed graft function**	0.484	1.622	1.142–2.305	**0.007**
**BKV viremia**	0.120	1.128	0.784–1.612	**0.517**
**Initial Steroids**	−0.148	0.862	0.250–2.974	**0.862**
**Initial MMF use**	1.120	3.065	1.051–8.937	**0.040**
**Initial CyA use**	−1.730	0.177	0.041–0.760	**0.020**
**Initial tacrolimus use**	1.730	5.643	1.316–24.200	**0.020**
**Initial mTor use**	−0.893	0.410	0.154–1.087	**0.073**
**Number of acute rejections**	0.256	1.292	1.117–1.495	**0.001**
**HLA-mismatches**	0.040	1.049	0.949–1.142	**0.390**

Bold: tested variables and *p*-values.

**Table 5:** Multivariable binary logistic regression.

Variable	Regression-Coefficient	Odds Ratio	95% CI	*p* Value
**Recipient age**	0.000	1.000	0.984–1.015	**0.959**
**Donor age**	0.020	1.020	1.006–1.034	**0.004**
**CMV match**	0.419	1.521	1.292–1.720	**<0.001**
**Living donation**	−0.614	0.541	0.351–0.834	**0.005**
**NODAT**	0.197	1.217	0.825–1.795	**0.197**
**DGF**	0.271	1.312	0.894–1.927	**0.165**
**Initial MMF use**	0.533	1.705	0.496–5.855	**0.397**
**Initial CyA use**	−2.059	0.128	0.021–0.766	**0.024**
**Number of acute rejections**	0.346	1.413	1.206–1.655	**<0.001**

Bold: tested variables and *p*-values.

## Supplementary Materials

The following are available online at https://www.mdpi.com/2077-0383/9/1/252/s1, Figure S1: Overall graft survival according to the development of a CMV-infection, Figure S2: Incidence of CMV viremia according to the CMV mismatch in the first year after transplantation.
