# Supplementary material for: Cytomegalovirus Viremia after Living and Deceased Donation in Kidney Transplantation
Source: J Clin Med. 2020 Jan 17;9(1):252. doi: 10.3390/jcm9010252 (PMC7019428; doi:10.3390/jcm9010252)
Supplement: Supplementary file 1 [file jcm-09-00252-s001.pdf]

Journal of
Clinical Medicine

MDPI

Supplementary Materials: The following are available online at www.mdpi.com/xxx/s1

Figure S1: Overall graft survival according to the development of a CMV-infection.

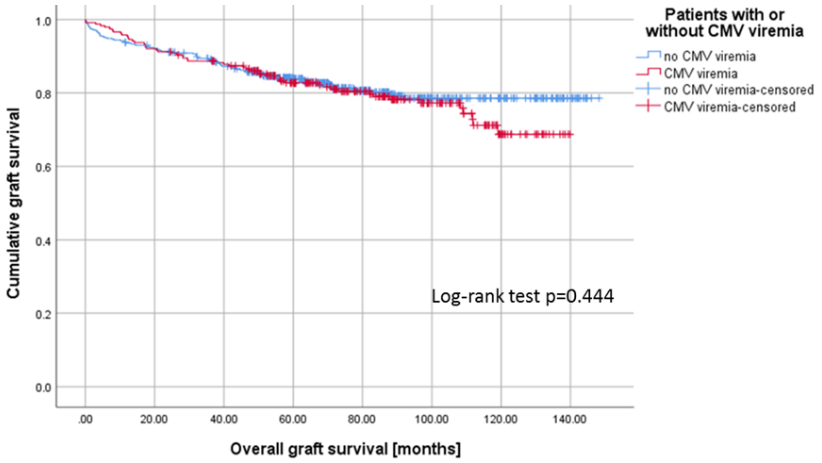

Patients-at-Risk

no CMV viremia	484	443	406	319	203	100	54	16
CMV viremia	239	220	205	163	119	72	24	0

Suppl. Figure 1: Kaplan Meier plot for overall graft survival, Log Rank: p=0.444 according to the development of a CMV-infection.

Figure S2: Incidence of CMV viremia according to the CMV mismatch in the first year after transplantation.

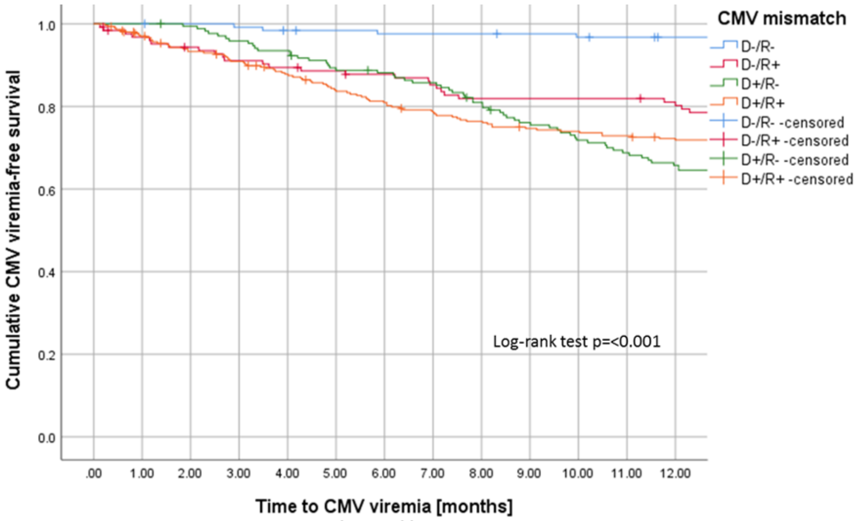

Patients-at-Risk

D-/R-	125	123	119	118	114
D-/R+	125	111	105	98	96
D+/R-	170	162	147	125	108
D+/R+	302	267	234	214	204

Suppl. Figure 2: Kaplan Meier plot for the incidence of CMV viremia according to the CMV mismatch in the first 12 months after transplantation in detail, Log Rank: p=<0.001.
